# Evolution of Male-Killer Suppression in a Natural Population

**DOI:** 10.1371/journal.pbio.0040283

**Published:** 2006-08-22

**Authors:** Emily A Hornett, Sylvain Charlat, Anne M. R Duplouy, Neil Davies, George K Roderick, Nina Wedell, Gregory D. D Hurst

**Affiliations:** 1 Department of Biology, University College London, London, United Kingdom; 2 Richard B. Gump South Pacific Research Station, University of California Berkeley, Maharepa, Moorea, French Polynesia; 3 School of Life Sciences, University of Queensland, Brisbane, Australia; 4 Centre for Ecology and Conservation, University of Exeter, Cornwall campus, Penryn, United Kingdom; University of Lausanne, Switzerland

## Abstract

Male-killing bacteria are widespread in arthropods, and can profoundly alter the reproductive biology of their host species. Here we detail the first case of complete suppression of a male killer. The nymphalid butterfly Hypolimnas bolina is infected with a strain of the bacterium *Wolbachia, w*Bol1, which kills male host embryos in Polynesian populations, but does not do so in many areas of Southeast Asia, where both males and female adults are naturally infected, and *w*Bol1-infected females produce a 1:1 sex ratio. We demonstrate that absence of male killing by *w*Bol1 is associated with dominant zygotic suppression of the action of the male killer. Simulations demonstrate host suppressors of male-killer action can spread very rapidly, and historical data indicating the presence of male killing in Southeast Asia in the very recent past suggests suppressor spread has been a very recent occurrence. Thus, male killer/host interactions are much more dynamic than previously recognised, with rapid and dramatic loss of the phenotype. Our results also indicate that suppression can render male killers completely quiescent, leading to the conclusion that some species that do not currently express a male killer may have done so in the past, and thus that more species have had their biology affected by these parasites than previously believed.

## Abstract 2

Within Southeast Asian populations, a nymphalid butterfly has recently evolved a zygotically acting suppressor that prevents the killing of its male offspring by a *Wolbachia* parasite.

## Introduction

Selfish genetic elements that distort the sex ratio of their host were first recorded over 80 y ago [1]. These elements provide seminal evidence that natural selection can occur at the level of the gene rather than the individual, the sex ratio distortion promoting the spread of the element, but at a fitness cost to most other genes of the bearer [2,3]. In creating sex ratio distortion, these elements increase the fitness of individuals producing the rare sex [4]. They therefore select for unlinked modifiers within the host genome that suppress the action or reduce the transmission efficiency of the sex ratio-distorting element, restoring the sex ratio to unity [5]. Evidence for this co-evolutionary “struggle” between sex ratio-distorting elements and their hosts is provided by studies of both sex chromosome meiotic drive [6] and cytoplasmic male sterility [7]. The majority of these sex ratio-distorting elements are found in the presence of host suppressors that inhibit their action [7,8].

Studies on meiotic drive and cytoplasmic male sterility contrast with those on male-killing bacteria/host interactions. Male killing is the most deleterious form of sex ratio distortion for the host, in which maternally inherited micro-organisms distort the sex ratio by killing male embryos thus producing the double fitness cost of mortality and failure to produce the rare sex. However, whilst male killers commonly achieve sufficient frequency within populations to cause significant fitness loss to their host, there is little evidence of evolved host responses to male-killing bacteria. Indeed, there is evidence that they can be maintained unsuppressed at high prevalence over long periods, at least in isolated oceanic island populations [9].

One interpretation of this observation is that suppression of male killing has evolved rarely. Alternatively, it is also possible that suppression evolves regularly, but the spread of the suppressor is rapid, and produces an infection that is quiescent and no longer easily recognizable as a male killer. This form of resistance is well known for other sex ratio distorters, with sex ratio distortion only being seen in inter-population crosses or in hybrids [10, 7]. Following this logic, extant male killers would simply be those that have not yet been suppressed.

We tested this idea in the tropical nymphalid butterfly Hypolimnas bolina. This species is found throughout the Indo-Pacific, providing a series of populations joined by restricted gene flow. A *Wolbachia* infection (strain *w*Bol1) is present in many populations. This strain expresses male killing in Polynesian populations [11]. However, the same strain of *Wolbachia* in Southeast Asian H. bolina is found in both males and females, and infected females produce a 1:1 sex ratio [12]. This inter-population variation is a good candidate for investigation as a potential case of host suppression of male killing within one part of a species range.

## Results

In the natural condition, *w*Bol1 exists in Hong Kong, Borneo, and Vietnam populations previously sampled, as well as in many Polynesian populations, including Moorea in French Polynesia [12]. In the three Southeast Asian populations, *w*Bol1 was found in all males sampled; in contrast, infection was found solely in females in Moorea, associated with its male-killing activity. Consistent with this, four wild females from Vietnam infected with *w*Bol1 were allowed to oviposit, each producing a 1:1 sex ratio with no male killing. Extensive crosses of *w*Bol1-infected females from Moorea indicate these always produce just females.

In the past 3 y, we have obtained four Thai and two Philippine stocks all derived independently from the wild, two generations ago. The origins of these stocks flank the previously observed populations to the East and West geographically, and all stocks displayed the same pattern as other Southeast Asian material: females were infected with *w*Bol1, eggs laid had a high egg hatch rate, and the broods produced had a 1:1 sex ratio, with resulting males infected.

In order to test for the role of host suppressor genes in causing phenotypic differences in *Wolbachia* infection, we investigated a) whether *w*Bol1 isolates from Moorea, known to be competent for male killing, retain or lose this ability on a Thai/Philippine nuclear genetic background; and, b) whether *w*Bol1 isolates from Thailand/Philippines, that currently do not kill males, do so when placed on a Moorean nuclear genetic background.

In each case the infection was placed onto the alternate nuclear genetic background via introgression. This was both technically easier than microinjection, and also had the added benefit of providing information concerning the genetic basis of any resistance.

### Do *w*Bol1 Isolates from Moorea Lose Male-Killing Ability on a Southeast Asian Nuclear Background?

In total, we examined the phenotype of four *w*Bol1 isolates collected from Moorea following crosses to males from Southeast Asia. These were compared to control crosses where the same *w*Bol1 isolate was crossed with males from within their native population. To allow the generality of any suppression to be ascertained, the different *w*Bol1 isolates were crossed to four different Southeast Asian lines in total. Two of the Moorean *w*Bol1 isolates were crossed to males from a single Philippine line (Phi '05), a third isolate to separate Philippine (Phi '06) and Thai lines (Thai '06), and a fourth to a distinct Thai line (Thai '05).

In each case, when infected Moorean females were mated to Thai or Philippine males, creating a nuclear background that was 50% Southeast Asian/50% Moorean, the females produced significant numbers of sons: male killing did not occur. This was in marked contrast with the natural condition of complete absence of males due to male killing (Table 1). The sex ratio deviates from the Moorean male killer controls where the isolates were mated “within-country” (contingency test: matriline 595 × Phi '05 male versus control:χ^2^ = 7.51, 1 d.f., *p* <0.01; matriline 787 × Thai '05 male versus control: χ^2^ = 4.44, 1 d.f., *p* <0.05; matriline 812 × Phi '05 male versus control: χ^2^ = 22.43, 1 d.f., *p* <0.001; matriline 1350 × Thai '06 male versus control:χ^2 ^ = 28.73, 1 d.f., *p* <0.001; matriline 1350 × Phi '06 male versus control: χ^2^ = 26.94, 1 d.f., *p* <0.001). The sex ratio in Moorea × Southeast Asia crosses was generally consistent with 1:1, with the exception of one replicate of the 787 × Thai '05. Egg hatch rates in these crosses were also consistent with absence of male killing in Moorea × Southeast Asia crosses and its presence in Moorea × Moorea controls (Table 1).

**Table 1:**
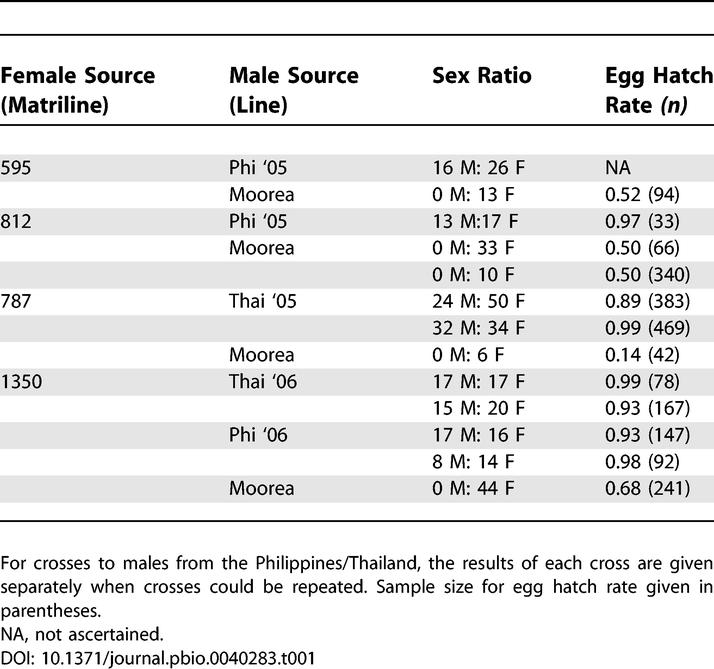
Sex Ratio and Egg Hatch Rates of Moorean Female Crosses with Philippine/Thai Males, Compared to Control Crosses with Wild-Type Moorean Males

A single generation of introgression of Southeast Asian nuclear genes was thus sufficient to suppress the male-killing activity of these *w*Bol1 isolates from Moorea. As a control to confirm the potency of these *w*Bol1 isolates, and to give some information about the genetic basis of the trait, we then attempted to recover male-killing ability in two isolates by backcrossing F1 females to wild males from their native Moorean population, re-introducing the Moorean nuclear background. We therefore backcrossed both 595 × Phi '05 and 787 × Thai '05 F1 females to Moorean males, and compared the sex ratio and egg hatch rate produced to that found on continued introgression of Phi '05 and Thai '05 nuclear genes respectively.

**Figure 1:**
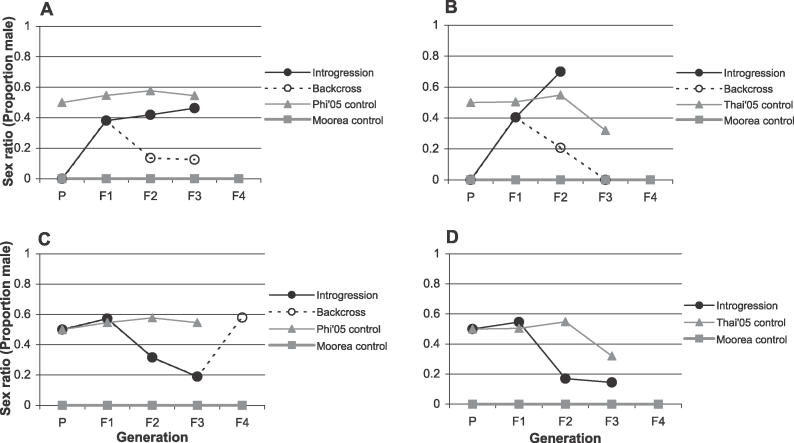
Sex Ratio Produced by *w*Bol1-Infected Females during Introgression onto Different Host Genetic Backgrounds (A) The mean sex ratio (proportion male) produced by *w*Bol1 infected H. bolina derived from the Moorean matriline 595 on introgression onto a Phi ‘05 nuclear background over three generations. Solid black: introgression onto a Phi ‘05 nuclear background; dashed black: backcross to Moorean males; grey triangles: Phi ‘05 control; grey squares: Moorean matriline 595 control. (B) As above, for the Moorean 787 *w*Bol1 isolate introgressed onto a Thai '05 nuclear background. Solid black: introgression; dashed black: backcross to Moorean males; grey triangles: Thai ‘05 control; grey squares: Moorean matriline 787 control. (C) The sex ratio (proportion male) produced by *w*Bol1 infected H. bolina derived from the Philippines (Phi '05) on introgression onto a Moorean nuclear background over 3 generations. Solid black: introgression; dashed black: backcross to Moorean males; grey triangles: Phi ‘05 control; grey squares: Moorean control. (D) As above, for the Thai ‘05 infection introgressed onto a Moorean nuclear background. Solid black: introgression; grey triangles: Thai '05 control; grey squares: Moorean control. Samples sizes and number of crosses performed are given in text and in Tables 1 and 2.

For Moorean females of line 595, previously crossed to Phi '05 males, results are given in Figure 1A. One generation of backcrossing to Moorean males produced a sex ratio in the progeny which immediately decreased from 29 males (M):43 females (F) (two crosses) to 6 M:38 F (one cross), and in a further generation of backcross the sex ratio was 8 M:69 F (two crosses). In contrast, continued introgression to Phi '05 males gave a 26 M:36 F sex ratio in the F2 (one cross) and 31 M:36 F in the F3 (two crosses), consistent with a 1:1 sex ratio (contingency tests: single generation of backcross versus F2 continued introgression: χ^2^ = 9.92, 1 d.f., *p* <0.01; second generation of backcross versus F3 continued introgression: χ^2^ = 23.33, 1 d.f., *p* <0.001; tests versus 1:1 sex ratio for continued introgression F2: χ^2^ =1.61, 1 d.f., NS; F3: χ^2^ =0.38, 1 d.f., NS). PCR assay proved all females to be *w*Bol1 positive at the end of the experiment.

**Table 2:**
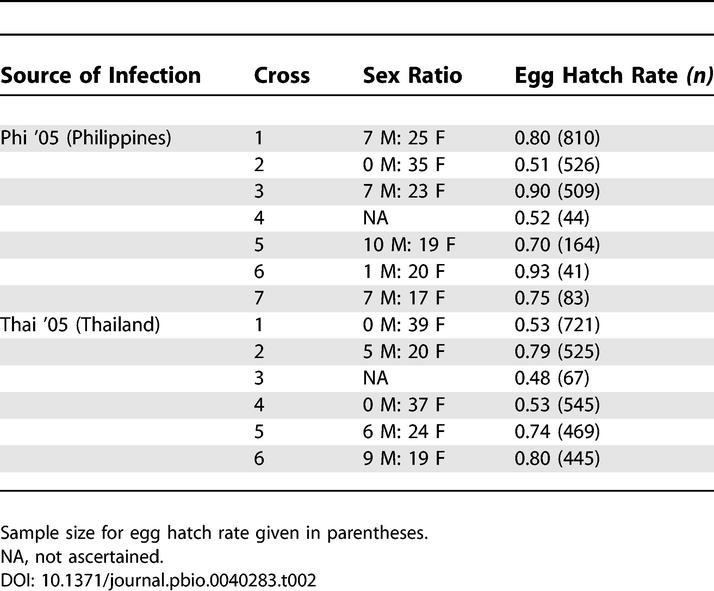
Sex Ratio and Egg Hatch Rates Produced in Generation Three of Introgression of the Infections from the Philippines and Thailand onto a Moorean Nuclear Background

For Moorean females from line 787 that had been crossed to Thai '05 males, results are given in Figure 1B. One generation of backcrossing moved the sex ratio from 56 M:84 F (two crosses) to 6 M:23 F (one cross) in the first generation of backcross. Continued introgression to Thai '05, in contrast, produced a sex ratio was 25 M:12 F in the F2 (two crosses) (contingency test: backcross versus continued introgression: χ^2^ = 14.42, 1 d.f., *p* <0.001; tests versus 1:1 sex ratio for continued introgression: χ^2^ = 4.57, 1 d.f., *p* <0.05). One generation of further backcross was achieved, producing 0 M:2 F; however, there was no comparator continued introgression. Again, PCR assay proved all females to be *w*Bol1 positive at the end of the experiment.

In both cases, egg hatch rates for the backcrosses to Moorean males decreases in line with the sex ratio, becoming “male killer-like,” in contrast to the egg hatch rates for the continued introgression onto Southeast Asian nuclear background. For line 595, the hatch rate produced on backcrosses to Moorean males gave hatch rates of 77% (two crosses) and then 62% (five crosses), compared to continued introgression of Phi '05, which produced hatch rates of 99% (two crosses) and 87% (three crosses) in the F2 and F3 respectively. Similarly, F1 females from the 787 female × Thai '05 male cross, produced an egg hatch rate of 72%, (three crosses) following the initial backcross to Moorean males, then an egg hatch rate of 55% (five crosses) in a further backcross, compared with 94% (six crosses) seen on continuing crossing to Thai '05 males.

### Do *w*Bol1 Isolates from Southeast Asia Kill Males When Placed on a Moorean Nuclear Background?

Concurrently with the above, we tested the ability of two *w*Bol1 isolates from Southeast Asia to induce male killing by examining the sex ratio produced by females infected with these *w*Bol1 isolates following introgression of nuclear genes from Moorea. Infected females from the Thai '05 and Phi '05 matrilines were thus independently mated with Moorean males from a variety of lines (these are presumed to all be non-suppressor, as there are no infected males in Moorea) over three generations, creating individuals with one of two Southeast Asian *w*Bol1 infections and progressively increasing Moorean nuclear background. These were compared to the same isolates maintained on their own genetic background.

Results from these crosses are given in Figures 1C and 1D. In the first generation, both females from Thai '05 and Phi '05 produced a sex ratio and egg hatch rate typical of their natural Southeast Asian phenotype. The Phi '05 × Moorean cross resulted in 39 M:29 F (four crosses), and the Thai '05 × Moorean cross resulted in 27 M:40 F (four crosses), neither of which differ from a 1:1 sex ratio (Philippine × Moorean: χ^2^ = 1.47, 1 d.f., NS; Thai × Moorean: χ^2^ = 2.5, 1 d.f., NS). Further introgression of Moorean nuclear genes produced a shift towards the male-killing condition. The sex ratio produced by Phi '05 infected females became more female biased, changing to 44 M:94 F (two crosses), in the F2 generation and 32 M:139 F (six crosses) in the F3 generation. Similarly, the sex ratio from Thai '05 infected females changed to 23 M:112 F (two crosses) in the F2, and 20 M:139 F (five crosses) in the F3. Continued introgression of Moorean nuclear genes did lead to a reduction in the number of males away from the controls (contingency test: a- the Philippines: F2 Phi '05 control versus Phi '05 × Moorea: χ^2^ = 11.39, 1 d.f., *p* <0.001; F3 Phi '05 control versus Phi '05 × Moorea: χ^2^ = 22.84, 1 d.f., *p* <0.001; b- Thailand: F2 Thai '05 control versus Thai '05 × Moorea: χ^2^ =24.82, 1 d.f., *p* <0.001; F3 Thai '05 control versus Thai '05 × Moorea: χ^2^ = 5.64, 1 d.f., *p* <0.025).

In accordance with the above, the egg hatch rate from the introgressions maintained the original state in the F1 (Philippine: 95% [eight crosses]; Thai: 98% [five crosses]), but moved towards that characteristic of male killing in the F2 and F3 (Philippine: F2 80% [seven crosses]; F3 74% [seven crosses]; Thai: F2 77% [three crosses]; F3 66% [six crosses]). These compared with the egg hatch rates produced from the Phi '05 controls (98.4%, ten crosses) and Thai '05 controls (81%, 12 crosses) which stayed in the natural condition.

During the third generation of introgression, a variety of independent crosses were performed as random Mendelian segregation of any nuclear suppressors is expected to have produced variability between lines at this stage, and would be informative as to the genetic basis of suppression (Table 2). Within these crosses, heterogeneity was indeed observed amongst both Philippine (χ^2^ = 17.84, 5 d.f., *p* <0.01) and Thai (χ^2^ = 23.44, 4 d.f., *p* <0.001) infections introgressed onto a Moorean nuclear background. Taking either lack of males or a 50% egg hatch rate as indicative of full male killing, five crosses were observed to exhibit full male killing, with no surviving males and 50% egg hatch rate. Eight other crosses exhibited partial male killing, with a female bias and a higher (about 75% or higher) egg hatch rate.

To confirm that the bacterium had not altered during introgression, F3 generation females from Phi '05 (from cross 2, as given in Table 2) were backcrossed to Southeast Asian males. Despite deriving from a fully male-killer sibship (0 M:35 F), this produced a sex ratio of 11 M:8 F, and was consistent with a 1:1 sex ratio (χ^2^ = 0.47, 1 d.f., NS). The egg hatch rate for this backcross increased correspondingly to 96% (two crosses). The backcross for Thai '05 failed to produce viable eggs. However, PCR assay confirmed that infection was maintained throughout all of the experiments above.

## Discussion

In the populations of H. bolina from Southeast Asia we have sampled, females carry *w*Bol1 infections, but these infections do not kill males. In contrast, *w*Bol1 infections in Polynesian populations of this species, such as Moorea, are active male killers. In this study, we demonstrate that the absence of male killing in Thai and Philippine lines is associated with suppression of the action of the male killer. Because Thailand and the Philippines geographically flank the areas formerly sampled (Hong Kong, Vietnam, and Borneo), suppression of male-killer activity is also the likely cause of male survival in these previously sampled areas.

Presence of a suppressor of male killing in Thailand and the Philippines is supported by two lines of evidence. First, *w*Bol1 isolates from Moorea in French Polynesia, known to kill males on their natural genetic background, do not kill males when the zygotes carrying this infection have a “Southeast Asian”/Moorean F1 hybrid nuclear background. Second, *w*Bol1 isolates from Thailand and the Philippines that do not express male killing on their natural background, do kill males when the matriline is serially crossed to males from Moorea. Male killing was first evident in the F2, and full male-killing activity was achieved in five of 13 cases in the F3. In both introgressions, re-introducing the original nuclear background by backcrossing reversed the trend observed through introgression, producing sex ratios and egg hatch rates more typical of the phenotype expressed in the infections' source populations.

In sum, these data demonstrate that the host genetic background found in populations of H. bolina from Southeast Asia can completely suppress the male-killing phenotype of *w*Bol1. The continued existence of *w*Bol1 in a non-male–killing state in wild males in Southeast Asia, and throughout our experiments, clearly demonstrates that this is a case of suppression of the action of the male killer rather than prevention of its transmission. Despite a lack of previous records, male killing is akin to cytoplasmic male sterility and meiotic drive, in that male killers can also select for nuclear genes that restore the sex ratio to 1:1.

We can also make inferences from our data about the mechanism and genetic basis of suppression. The survival of males in the crosses between Moorean females and Southeast Asian males leads us to conclude that suppression occurs zygotically: male survival occurs despite a maternal genotype that allows male killing. The individuals that survive here are F1 hybrids, and are effectively “Southeast Asia”/Moorea heterozygotes across their nuclear genome. That these male heterozygotes survive at near wild-type rates indicates that the zygotic suppressor is genetically dominant and has high penetrance. This conclusion is confirmed by the lack of change in sex ratio in the first generation of the reciprocal “Southeast Asian” female × Moorean male crosses. Here, the first generation creates heterozygous hybrid males as above, which survive. Only in the F2, when segregation of the suppressor gene/genes occurs such that 50% of loci will be “pure Polynesian” do we see the emergence of male killing. The dominance of the suppressor also parallels the case for restorers of cytoplasmic male sterility and meiotic drive [7, 8].

The observations are compatible with suppression being controlled at a single locus. For a single locus and dominant suppression, introgression of the *w*Bol1 isolates from Southeast Asia onto a Moorean nuclear background would be expected to produce a 1:1 sex ratio in the F1 and two females per male in the F2 (half of the males die as they lack the suppressor). In the F3, 50% of crosses would be expected to produce a 2:1 sex ratio and 50% full male killing, associated with previous segregation of the gene. We do see a 2:1 sex ratio in the F2, and five of 13 crosses in the F3 show full male killing, and our data is statistically compatible with this null hypothesis. In comparison, a model with two loci where either locus is sufficient for rescue can be statistically rejected (this would create 4:3 sex ratios in both the F2 and in 25% of F3 crosses, neither of which are observed), as can a model with two loci where both are required (this would produce 75% of crosses having full male killing in the F3, which deviates from our observed five of 13 crosses). A single dominant locus thus is the best simple explanation of our data.

**Figure 2:**
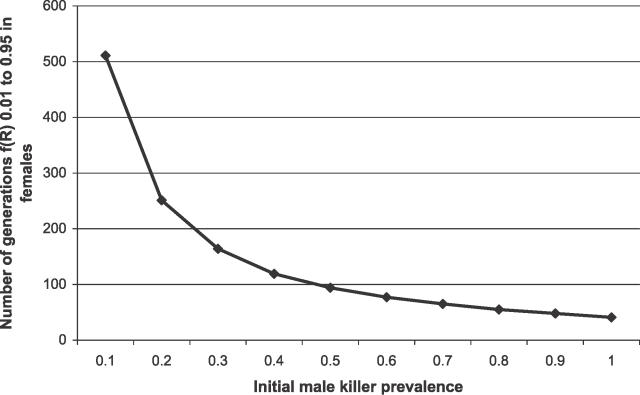
Time of Spread of the Male-Killing Suppressor with Varying Initial Male Killer Prevalence Time (given as the number of host generations) taken to spread from 1% to 95% frequency for a zygotically acting, single locus dominant suppressor of male killing, with varying initial prevalence of male-killing *w*Bol1 strain. Results are derived from simulation of gene frequency changes for varying initial prevalence of *w*Bol1 (given in Protocol S1), with the assumption that *w*Bol1 shows perfect vertical transmission and no direct benefit or cost to the female host, save death of male offspring. The simulation assumes the suppressor rescues males completely, is fully penetrant, and has no direct cost. Code for the recursions is available on request.

In addition, we can infer the speed with which the suppressor we discovered spreads in natural populations. For any initial prevalence of the male killer in excess of 0.5, the suppressor would spread from 1% to 95% frequency in less than 100 generations (Figure 2, Protocol S1). This assumes that the male killer is perfectly efficient at killing males, is maintained at its current frequency despite losing male-killing ability (an empirical observation [12]), and a dominant zygotically acting suppressor of male killing that is selectively neutral save the ability to rescue males. These assumptions being robust, loss of the male-killing phenotype will occur very rapidly following the initial suppressor mutation, rendering the male killer quiescent. Past data indicates that the male killer was common in some populations of Southeast Asia, with around 80–90% of females carrying the male killer in Borneo [13]. This would produce a population sex ratio of five to ten females per male, making male survival a highly selected trait spreading to 95% frequency inside 48 generations (for 90% initial prevalence) and 55 generations (for 80% initial prevalence).

The historical record also suggests that this rapid spread is likely to have been a very recent process. Whilst the male-killing phenotype does not occur in Borneo currently (*w*Bol1 is present in all males collected in recent samples [12]), it was present in Borneo H. bolina in the 1960s (160 generations ago) [13]. The inference of recent spread of the suppressor assumes that past samples from Borneo are comparable with present samples, despite being taken from locations approximately 500 km apart, and that occurrence of infected males in Borneo represents presence of the same suppressor elements demonstrated in Philippine/Thai populations. Whilst not proven, both these factors are unlikely to matter in a highly migratory species such as H. bolina [14], and our data are clearly compatible with a very recent episode of selection for suppression. We would also predict that this suppressor will, in the near future, spread across Polynesian H. bolina following rare dispersal events, and that, in the absence of counter-adaptation by *w*Bol1, male killing will cease in this species within the lifetime of a human observer.

We have demonstrated that male killers can be suppressed within a natural population. Previously, the high numbers of arthropod species infected with male killers has been regarded as a function of a slow rate of loss from infected species (death), rather than a high rate of initial establishment and spread (birth) coupled with rapid loss. If suppressor spread is common (rapid loss), then the incidence of male killers would reflect a higher “birth rate” of new interactions than previously imagined. The appearance of male killing following transinfection of *Wolbachia* strain *w*CauA from the moth *Cadra cautella,* where the bacteria does not produce this phenotype, to the closely related *Ephestia kuehniella,* is certainly consistent with such a scenario [15,16]. Future ascertainment of the frequency of suppressed male killing in other species will allow precise estimation of the dynamics of these fascinating parasites.

## Materials and Methods

Six independent replicate Hypolimnas bolina stocks from Southeast Asia were obtained from pupae suppliers in the United Kingdom (Thai pupae from Stratford-upon-Avon Butterfly Farm, and Philippine pupae from London Pupae Supplies). These stocks each represent F2 progeny from wild caught females from the area indicated (note, export and import regulations requires the exported material to have been captive bred for two generations), and derive from different founder individuals. Five of these stocks were sent to Moorea under permit, and bred in Moorea. A further stock was bred in the United Kingdom from pupae previously obtained. While the stocks obtained are each likely to be internally heterogeneous, they come without breeding history, and thus each is conservatively treated as a single line to avoid pseudoreplication.

Adults were individually marked on their wings to permit identification, and crosses conducted in a large outdoor flight cage. Following mating, oviposition was encouraged by placing the female into a clear container also containing a young Syndrella nodiflora plant. A well-lit situation was required for optimal oviposition. On day 4 after oviposition, the eggs develop and hatch. On day 5 the number of green eggs (infertile eggs), brown eggs (developed but unhatched eggs), and hatched eggs (1^st^ instar larvae) were counted in each clutch. The egg hatch rate (HR) is calculated as follows: HR = *n* (hatched eggs) / *n* (hatched eggs) + *n* (brown eggs). Larvae were reared inside a laboratory under natural light and temperatures and fed on an excess of *Asystasia gangetica,* a larval host plant found in Moorea. Sex ratio was recorded on emergence as adult.

PCR assays for *w*Bol1 presence were based on *Wolbachia*-specific PCR primers, 81F and 522R that amplify the *wsp* gene of B-clade *Wolbachia* [17]. Prior to assay for *Wolbachia,* the primer pair CO1F and CO1R were used to confirm the quality of the DNA extraction [18].

## Supporting Information

Protocol S1 Derivation of Recursions to Calculate the Speed of Spread of a Zygotically Acting Dominant Suppressor of Male Killing, with Varying Male-Killer Frequency Simulations were initialized with no infected males in the population, the suppressor gene present in heterozygous females only, with equal relative frequency in the infected and uninfected population.
(169 KB PDF)
