# Supplementary material for: Evolution of Male-Killer Suppression in a Natural Population
Source: PLoS Biol. 2006 Aug 22;4(9):e283. doi: 10.1371/journal.pbio.0040283 (PMC1551922; doi:10.1371/journal.pbio.0040283)
Supplement: Supplementary file 1 [file pbio.0040283.sd001.pdf]

Protocol S1. Derivation of recursions to calculate the speed of spread of a zygotically acting dominant suppressor of male killing, with varying male killer frequency. Simulations were initialized with no infected males in the population, the suppressor gene present in heterozygous females only, with equal relative frequency in the infected and uninfected population.

Class, type and frequency of individual:

Classes of individual	Type	Frequency
Male infected with wBol1 and homozygous for the suppression gene	*ImRR*	M1
Male infected with wBol1 and heterozygous for the suppression gene	*ImRr*	M2
Male uninfected with wBol1 and homozygous for the suppression gene	*UmRR*	M3
Male uninfected with wBol1 and heterozygous for the suppression gene	*UmRr*	M4
Male uninfected with wBol1 and homozygous for the wild-type gene	*Umrr*	M5
Female infected with wBol1 and homozygous for the supression gene	*IfRR*	F1
Female infected with wBol1 and heterozygous for the suppression gene	*IfRr*	F2
Female infected with wBol1 and homozygous for the wild-type gene	*Ifrr*	F3
Female uninfected with wBol1 and homozygous for the suppression gene	*UfRR*	F4
Female uninfected with wBol1 and heterozygous for the suppression gene	*UfRr*	F5
Female uninfected with wBol1 and homozygous for the wild-type gene	*Ufrr*	F6

Note: Type *Imrr* is not included in this model as the individuals with this make-up would not survive- being killed by the male killing action of wBol1 as there is no suppression gene

Crossing scheme: progeny types from all cross combinations, assuming Mendelian segregation of the suppressor, perfect vertical transmission, and death of infected males lacking the suppressor.

Females \ Males		M1 *ImRR*		M2 *ImRr*		M3 *UmRR*		M4 *UmRr*		M5 *Umrr*	
**F1**	*IfRR*	ImRR	IfRR	0.5 ImRR<br>0.5 ImRr	0.5 IfRR<br>0.5 IfRr	ImRR	IfRR	0.5 ImRR<br>0.5 ImRr	0.5 IfRR<br>0.5 IfRr	ImRr	IfRr
**F2**	*IfRr*	0.5 ImRR<br>0.5 ImRr	0.5 IfRR<br>0.5 IfRr	0.25 ImRR<br>0.5 ImRr	0.25 IfRR<br>0.25 Ifrr<br>0.5 IfRr	0.5 ImRR<br>0.5 ImRr	0.5 IfRR<br>0.5 IfRr	0.25 ImRR<br>0.5 ImRr	0.25 IfRR<br>0.25 Ifrr<br>0.5 IfRr	0.5 ImRr	0.5 Ifrr<br>0.5 IfRr
**F3**	*Ifrr*	ImRr	IfRr	0.5 ImRr	0.5 Ifrr<br>0.5 IfRr	ImRr	IfRr	0.5 ImRr	0.5 Ifrr<br>0.5 IfRr	0	Ifrr
**F4**	*UfRR*	UmRR	UfRR	0.5 UmRR<br>0.5 UmRr	0.5 UfRR<br>0.5 UfRr	UmRR	UfRR	0.5 UmRR<br>0.5 UmRr	0.5 UfRR<br>0.5 UfRr	UmRr	UfRr
**F5**	*UfRr*	0.5 UmRR<br>0.5 UmRr	0.5 UfRR<br>0.5 UfRr	0.25 UmRR<br>0.5 UmRr<br>0.25 Umrr	0.25 UfRR<br>0.25 Ufrr<br>0.5 UfRr	0.5 UmRR<br>0.5 UmRr	0.5 UfRR<br>0.5 UfRr	0.25 UmRR<br>0.5 UmRr<br>0.25 Umrr	0.25 UfRR<br>0.25 Ufrr<br>0.5 UfRr	0.5 UmRr<br>0.5 Umrr	0.5 Ufrr<br>0.5 UfRr
**F6**	*Ufrr*	UmRr	UfRr	0.5 UmRr<br>0.5 Umrr	0.5 Ufrr<br>0.5 UfRr	UmRr	UfRr	0.5 UmRr<br>0.5 Umrr	0.5 Ufrr<br>0.5 UfRr	Umrr	Ufrr

Legend:

**I**	Infected
**U**	Uninfected
**m**	Male
**f**	Female
**R**	Dominant suppression gene
**r**	Recessive wild-type gene

Recursions:

**M1' =**	[(M1 X F1) + 0.5(M2 X F1) + (M3 X F1) + 0.5(M4 X F1) + 0.5(M1 X F2) + 0.25(M2 X F2) + 0.5(M3 X F2) + 0.25(M4 X F2)]/Mnorm
**M2' =**	[0.5(M2 X F1) + 0.5(M4 X F1) + (M5 X F1) + 0.5(M1 X F2) + 0.5(M2 X F2) + 0.5(M3 X F2) + 0.5(M4 X F2) + 0.5(M5 X F2) + (M1 X F3) + 0.5(M2 X F3) + (M3 X F3) + 0.5(M4 X F3)]/Mnorm
**M3' =**	[(M1 X F4) + 0.5(M2 X F4) + (M3 X F4) + 0.5(M4 X F4) + 0.5(M1 X F5) + 0.25(M2 X F5) + 0.5(M3 X F5) + 0.25(M4 X F5)]/Mnorm
**M4' =**	[0.5(M2 X F4) + 0.5(M4 X F4) + (M5 X F4) + 0.5(M1 X F5) + 0.5(M2 X F5) + 0.5(M3 X F5) + 0.5(M4 X F5) + 0.5(M5 X F5) + (M1 X F6) + 0.5(M2 X F6) + (M3 X F6) 0.5(M4 X F6)]/Mnorm
**M5' =**	[0.25(M2 X F5) + 0.25(M4 X F5) + 0.5(M5 X F5) + 0.5(M2 X F6) + 0.5(M4 X F6) + (M5 X F6)]/Mnorm
**Mnorm =**	∑ numerators above
**F1' =**	[(M1 X F1) + 0.5(M2 X F1) + (M3 X F1) + 0.5(M4 X F1) + 0.5(M1 X F2) + 0.25(M2 X F2) + 0.5(M3 X F2) + 0.25(M4 X F2)]/Fnorm
**F2' =**	[0.5(M2 X F1) + 0.5(M4 X F1) + (M5 X F1) + 0.5(M1 X F2) + 0.5(M2 X F2) + 0.5(M3 X F2) + 0.5(M4 X F2) + 0.5(M5 X F2) + (M1 X F3) + 0.5(M2 X F3) + (M3 X F3) + 0.5(M4 X F3)]/Fnorm
**F3' =**	[0.25(M2 X F2) + 0.25(M4 X F2) + 0.5(M5 X F2) + 0.5(M2 X F3) + 0.5(M4 X F3) + (M5 X F3)]/Fnorm
**F4' =**	[(M1 X F4) + 0.5(M2 X F4) + (M3 X F4) + 0.5(M4 X F4) + 0.5(M1 X F5) + 0.25(M2 X F5) + 0.5(M3 X F5) + 0.25(M4 X F5)]/Fnorm
**F5' =**	[0.5(M2 X F4) + 0.5(M4 X F4) + (M5 X F4) + 0.5(M1 X F5) + 0.5(M2 X F5) + 0.5(M3 X F5) + 0.5(M4 X F5) + 0.5(M5 X F5) + (M1 X F6) + 0.5(M2 X F6) + (M3 X F6) + 0.5(M4 X F6)]/Fnorm
**F6' =**	[0.25(M2 X F5) + 0.25(M4 X F5) + 0.5(M5 X F5) + 0.5(M2 X F6) + 0.5(M4 X F6) + (M5 X F6)]/Fnorm
**Fnorm =**	∑ numerators above
